# Symptoms and pathogens diversity of Corn *Fusarium* sheath rot in Sichuan Province, China

**DOI:** 10.1038/s41598-021-82463-2

**Published:** 2021-02-02

**Authors:** Wei Wang, Bo Wang, Xiaofang Sun, Xiaobo Qi, Conghao Zhao, Xiaoli Chang, Muhammad Ibrahim Khaskheli, Guoshu Gong

**Affiliations:** 1 College of Agronomy, Sichuan Agricultural University, Chengdu, 611130 China; 2 Department of Plant Protection, Faculty of Crop Protection, Sindh Agriculture University, Tandojam, 70060 Pakistan

**Keywords:** Microbiology, Diseases

## Abstract

To elucidate the symptoms and pathogens diversity of corn *Fusarium* sheath rot (CFSR), diseased samples were collected from 21 county-level regions in 12 prefecture-level districts of Sichuan Province from 2015 to 2018 in the present study. In the field, two symptom types appeared including small black spots with a linear distribution and wet blotches with a tawny or brown color. One hundred thirty-seven *Fusarium* isolates were identified based on morphological characteristics and phylogenetic analysis (*EF1-α*), and Koch’s postulates were also assessed. The results identified the isolates as 8 species in the *Fusarium* genus, including *F. verticillioides*, *F. proliferatum*, *F. fujikuroi*, *F. asiaticum*, *F. equiseti*, *F. meridionale*, *F. graminearum* and *F. oxysporum*, with isolation frequencies of 30.00, 22.67, 15.33, 7.33, 6.00, 5.33, 3.33 and 1.33%, respectively. *Fusarium* *verticillioides* and *F. proliferatum* were the dominant and subdominant species, respectively. Two or more *Fusarium* species such as *F. verticillioides* and *F. proliferatum* were simultaneously identified at a mixed infection rate of 14.67% in the present study. The pathogenicity test results showed that *F. proliferatum* and *F. fujikuroi* exhibited the highest virulence, with average disease indices of 30.28 ± 2.87 and 28.06 ± 1.96, followed by *F. equiseti* and *F. verticillioides*, with disease indices of 21.48 ± 2.14 and 16.21 ± 1.84, respectively. *Fusarium asiaticum*, *F. graminearum* and *F. meridonale* showed lower virulence, with disease indices of 13.80 ± 2.07, 11.57 ± 2.40 and 13.89 ± 2.49, respectively. Finally, *F. orysporum* presented the lowest virulence in CFSR, with a disease index of 10.14 ± 1.20. To the best of our knowledge, this is the first report of *F. fujikuroi*, *F. meridionale* and *F. asiaticum* as CFSR pathogens in China.

## Introduction

Maize (*Zea mays* L.) is one of the largest food staples worldwide and is one of the most economically important crops in China^1^. Maize yields as high as 260.77 billion kilograms have been attained in China, greatly contributing to ensuring food safety and increasing farmer income^2–4^. In China, the maize-sowing area reached approximately 44.96 million hm^2^ in 2015, after which it decreased to 42.39 million hm^2^ in 2017^5^. Chinese maize production of 259 million tons has been reported, accounting for 39% of Chinese cereal crop production and 22.8% of the global maize output^6^. However, several diseases caused by fungi, bacteria and viruses are a major factor limiting maize production. Among these diseases, *Fusarium* spp. can cause ear rot, stalk rot, seedling blight and root rot^7^.

Corn *Fusarium* sheath rot (CFSR) is one of the most serious crop diseases in China^8,9^. The results of previous studies suggest that *Fusarium proliferatum* can not only affect maize production but also produces the toxin fumonisins, posing great risks to human and livestock^10^. *F. graminearum*, *F. verticillioides* and *F. equiseti* have also been identified as causal agents of CFSR^8,11^. In fields, these pathogens primarily infect the sheath from the late growth period to the grain-forming stage^11^. Initial symptoms appear as irregular circular brown necrotic spots, after which the entire sheath gradually appears water-soaked and finally dies^12,13^. A severely infected sheath can eventually reduce lodging resistance and yield. According to statistical data, lodging results in major economic losses of approximately 15–25% and can even result in total crop failure^14^. A positive correlation has been demonstrated between yield loss and disease severity^15^. Additionally, wounds caused by aphid feeding can exacerbate the sheath rot severity of maize^16^.

Studies have shown that CFSR has occurred in more than 12 provinces in China^17^. However, the disease has not been reported in Sichuan Province, a major maize-producing region in China. Therefore, the goal of our present study was to characterize the disease severity, symptoms and *Fusarium* spp. pathogens of CFSR, which will provide an important basis for effective integrated control of this disease.

## Results

### Occurrence of corn *Fusarium* sheath rot (CFSR) in the field

**Figure 1:**
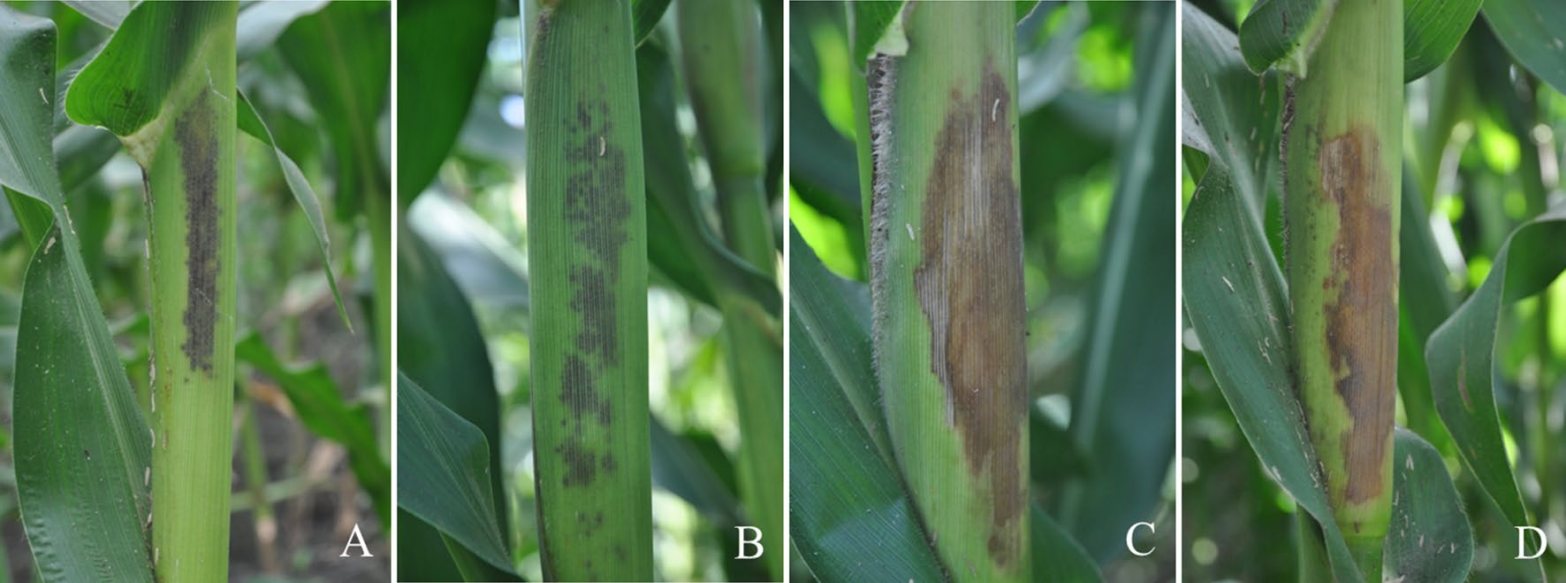
Disease symptoms of corn *Fusarium* sheath rot in the field were divided into two types of symptoms: small black spots with a linear distribution (**A**,**B**) and wet blotches with a tawny or brown color (**C**,**D**).

**Figure 2:**
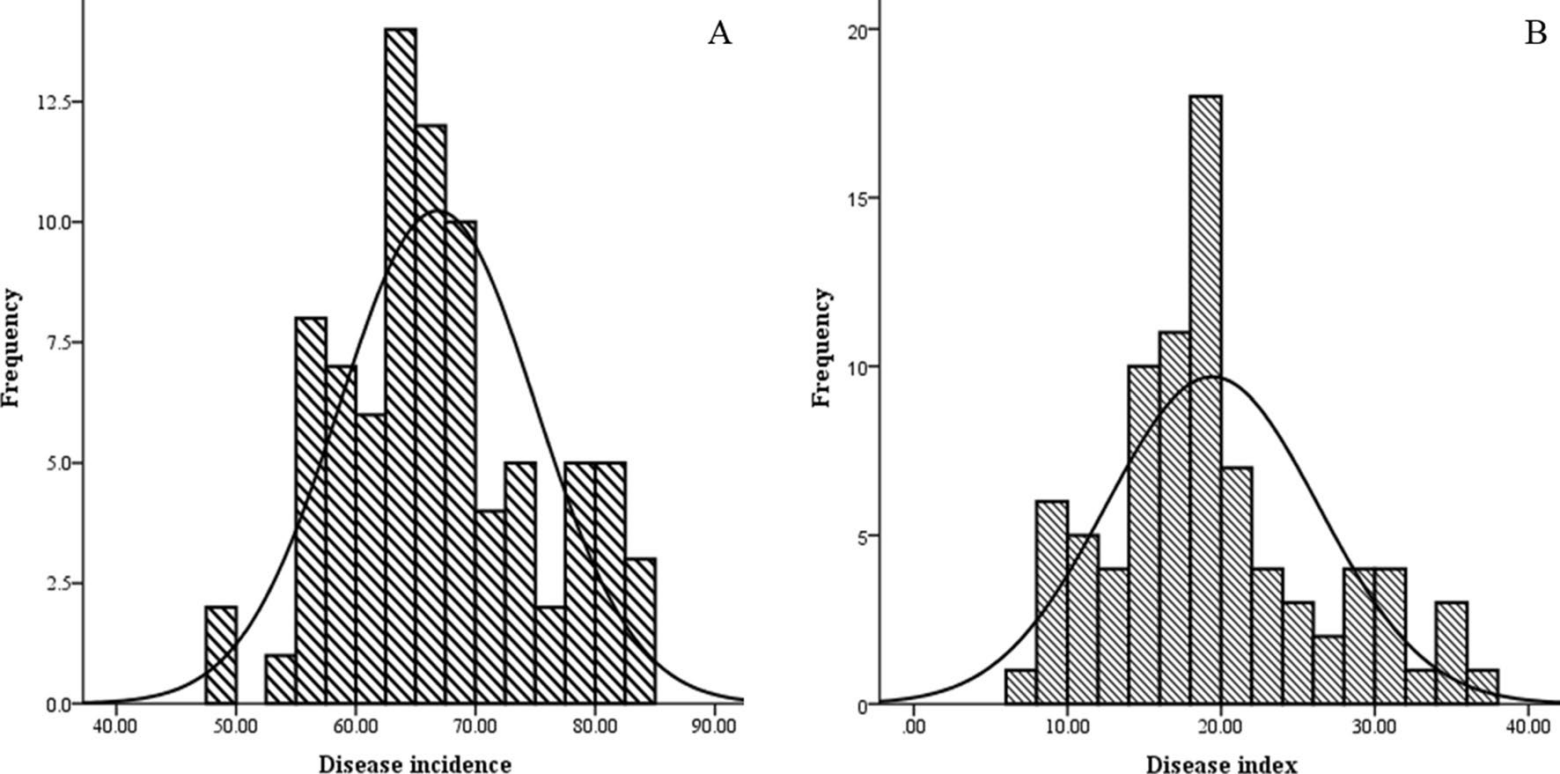
Statistics of disease incidence (**A**) and the disease index (**B**) of corn *Fusarium* sheath rot at 84 investigated sites in Sichuan Province, China.

In the present study, we investigated the occurrence of CFSR from 2015 to 2018. CFSR is characterized by two primary types of disease symptoms in the field (Fig. 1), including small black spots with a linear distribution (Fig. 1A,B) and wet blotches with a tawny or brown color (Fig. 1C,D). Disease incidence primarily ranged from 55 to 70% (Fig. 2A), and the disease index primarily ranged from 14.00 to 22.00 for CFSR (Fig. 2B). Among 84 investigated spots, the disease incidence ranged from 49.59–84.16%, with an average of 66.83 ± 0.89%, while the disease index ranged from 7.32 to 37.62, with an average of 19.42 ± 0.75 (Table 1).

**Table 1:** Statistical description of the disease incidence and disease index values based on the investigation of corn *Fusarium* sheath rot in the field.

	N	Minimum	Maximum	Standard deviation	Average		Skewness		Kurtosis	
					Statistic	SE	Statistic	SE	Statistic	SE
Disease index	84	7.32	37.62	6.92	19.42	0.75	0.62	0.26	0.004	0.52
Disease incidence	84	49.59%	84.18%	8.19%	66.83%	0.89%	0.29	0.26	-0.43	0.52

### Identification of *Fusarium* species associated with CFSR

**Figure 3:**
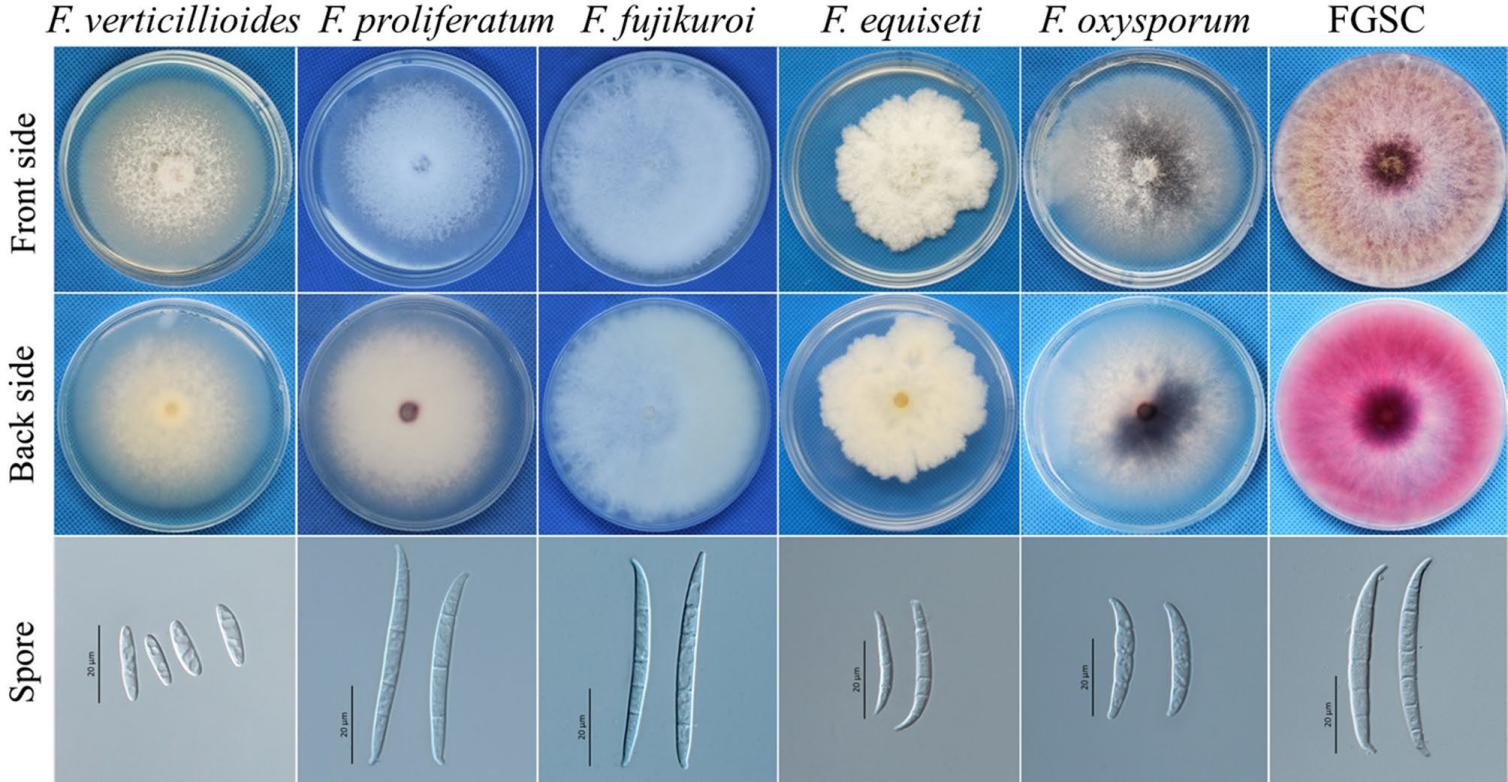
Representative colonies formed on PDA and conidial morphology characteristics.

One hundred thirty-seven *Fusarium* isolates from 150 maize sheath samples were divided into six types according to the color and shape of their colonies and the morphology of their conidia (Fig. 3). The features of the macroconidia are described in Table 2. For further molecular verification, partial *rDNA-ITS* gene sequences were amplified, generating a 560-bp band, and analyses of sequence similarity showed that 137 *Fusarium* isolates exhibited greater than 95–99% similarity with sequences from the *F. graminearum* species complex (FGSC), *F. fujikuroi* species complex (FFSC), *F. incarnatum-equiseti* species complex (FIESC), *F. oxysporum* and *F. verticillioides* in the databases of NCBI (http://www.ncbi.nlm.nih.gov) and the FUSARIUM-ID (http://isolate.fusariumdb.org/guide.php).

**Table 2:** The morphological characteristics of *Fusarium* species were observed on PDA for 3 days at approximately 25 °C under 12 h of light per day.

Groups	Colonies appearance	Growth rate (mm/day)	Conidia				
			Length (μm)	Width (μm)	Septum	Shape	Foot spore
*F. verticillioides*	White mycelia and light yellow regularly colony	4.76 ± 0.57b	23.58 ± 2.81e	3.74 ± 0.20bc	3–4	Fusiform	+
*F. proliferatum*	White mycelia and purple regularly colony	6.47 ± 0.62a	45.72 ± 5.14c	3.55 ± 0.27c	3–4	Fusiform, Falcate	+
*F. fujikuroi*	White mycelia and offwhite regularly colony	6.43 ± 0.60a	52.17 ± 2.13ab	4.09 ± 0.19b	3–5	Falcate	+
*F. equiseti*	White mycelia and light yellow unregularly colony	4.92 ± 0.56b	18.34 ± 2.94f.	4.14 ± 0.95b	2–3	Matt, Falcate	−
*F. oxysporum*	White mycelia and modena regularly colony	5.03 ± 1.14b	28.43 ± 4.29d	4.08 ± 0.67b	2–3	Falcate	+
FGSC	White and yellow mycelia and red regularly colony	6.64 ± 0.42a	50.98 ± 6.45b	4.96 ± 0.34a	4–6	Falciform	+

Different lowercase letters indicate a significant difference at the 5% level by Duncan’s least significant range test. FGSC: *Fusarium graminearum* species complex.

**Figure 4:**
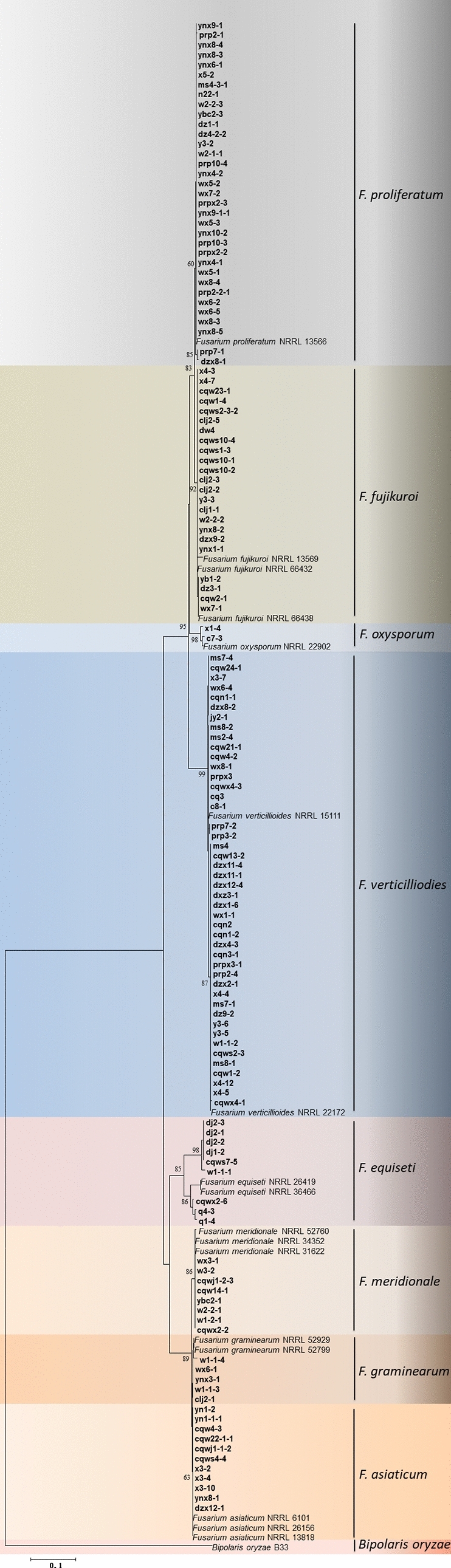
Phylogenetic tree of *Fusarium* isolates based on neighbor-joining analysis of the *EF1-α* gene; bootstrap values are from a bootstrap test of 1000 replicates. Isolates for which type strains were included in the study are indicated in boldface.

In addition, a partial *EF-1α* gene sequence was amplified, generating a 700-bp band. For further phylogenetic analysis, a neighbor-joining tree based on the *EF-1α* gene was constructed, which included 137 *Fusarium* isolates, 17 reference isolates and 1 outgroup isolate of *Bipolaris oryzae* (B33, KJ 939510) (See Supplementary Table S1 online). As shown in Fig. 4, all isolates were clearly classified into eight species, including *F. vertieillioides*, *F. proliferatum*, *F. fujikuroi*, *F. asiaticum*, *F. equiseti*, *F. meridonale*, *F. graminearum* and *F. orysporum*.

### Isolation frequency of eight *Fusarium* species

**Figure 5:**
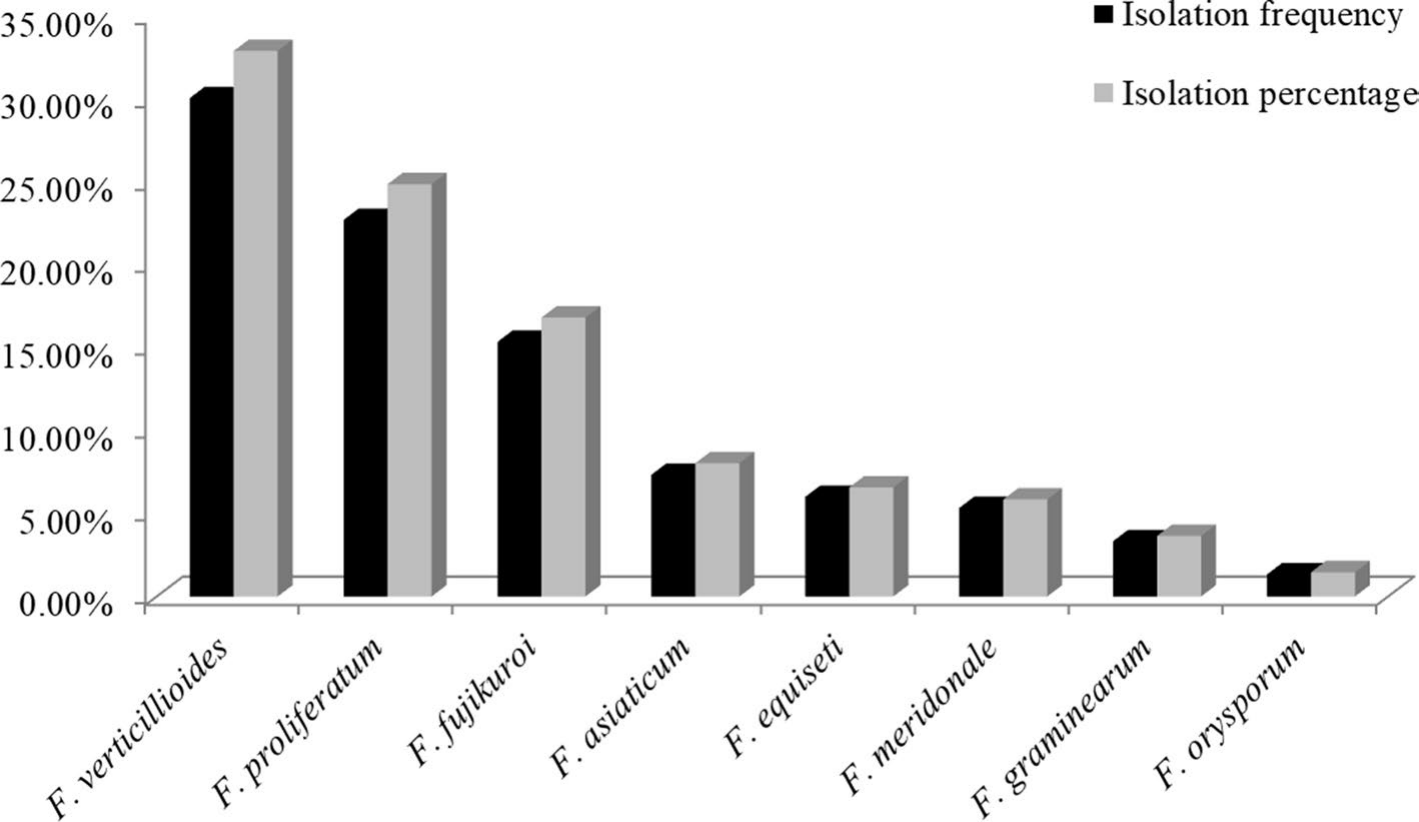
Isolation frequency of *Fusarium* species from maize sheaths in Sichuan Province, China.

In the present study, *F. verticillioides* and *F. proliferatum* were always observed in mixed infections on maize sheaths. Two or more *Fusarium* species were simultaneously identified from each of 22 sheath samples with a mixed infection rate 14.67%, and the frequencies at which *Fusarium* species were isolated are shown in Fig. 5 and Table S2. The isolation frequencies of *F. verticillioides*, *F. proliferatum*, *F. fujikuroi*, *F. asiaticum*, *F. equiseti*, *F. meridionale*, *F. graminearum* and *F. oxysporum* were 30.00, 22.67, 15.33, 7.33, 6.00, 5.33, 3.33 and 1.33%, respectively. Additionally, a comparison of the percentages of isolates obtained for the eight *Fusarium* species revealed that *F. verticillioides* accounted for 32.84% of all *Fusarium* isolates, followed by 24.82% for *F. proliferatum*, 16.79% for *F. fujikuroi*, 8.03% for *F. asiaticum*, 6.57% for *F. equiseti* and 5.84% for *F. meridonale*, while *F. graminearum* and *F. orysporum* accounted for 3.65 and 1.46% of the isolates, respectively.

### Pathogenicity test of *Fusarium* species

**Figure 6:**
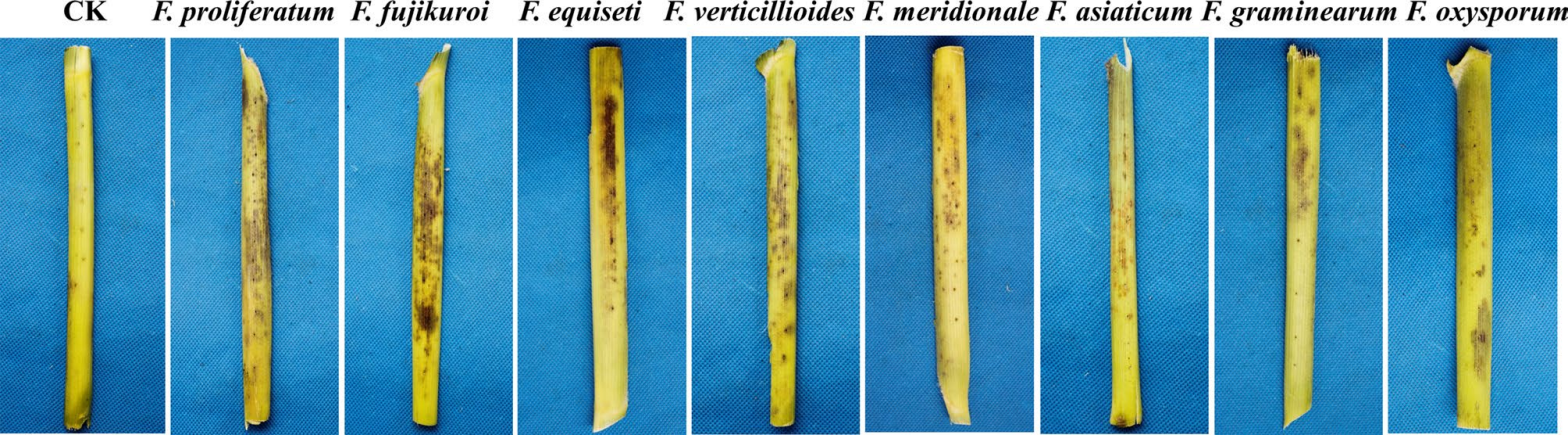
Inoculation of maize sheaths with different *Fusarium* spp. isolates (the maize cultivar is Chuandan 428).

To assess the pathogenicity of the *Fusarium* species identified from the maize sheaths in Sichuan Province, symptoms of CFSR were observed at 25 days after inoculation with twenty-three representative *Fusarium* isolates from eight *Fusarium* species (Table 2), and the disease index was calculated according to disease severity caused by *Fusarium* species. Four maize cultivars were tested here. The symptoms of small black spots with a homogeneous distribution (caused by *F. proliferatum*, *F. fujikuroi*, *F. equiseti*, *F. verticillioides*, *F. meridonale*, *F. asiaticum* and *F. graminearum*) and wet blotches with a tawny color (caused by *F. orysporum*) were observed after inoculation, while the control plants showed no significant symptoms (Fig. 6).

All *Fusarium* isolates were pathogenic and caused CFSR, with disease indices ranging from 10.14–30.28. Among the assayed isolates, *F. proliferatum* and *F. fujikuroi* showed significantly higher virulence than the other *Fusarium* species (P < 0.05), with average disease indices of 30.28 ± 2.87 and 28.06 ± 1.96 (Table 3), followed by *F. equiseti* and *F. verticillioides*, which had similar disease indices of 21.48 ± 2.14 and 16.21 ± 1.84, respectively. *F. asiaticum*, *F. graminearum* and *F. meridonale*, members of the FGSC, also caused CFSR following inoculation but with somewhat lower virulence, with disease indices ranging from 11.57–13.89. *F. orysporum* showed the lowest virulence, causing CFSR with a disease index of 10.14 ± 1.20. For this species, there were no sheath rot symptoms and only a few signs of mechanical damage on the noninoculated maize sheath (Fig. 6). Finally, the pathogens were reisolated and identified, applying Koch’s postulates to determine their pathogenicity. Our results demonstrated that the disease symptoms of wet blotches with a tawny or brown color were caused by *F. orysporum*, whereas small black spots were caused by *F. verticillioides*, *F. proliferatum*, *F. equiseti*, *F. asiaticum*, *F. graminearum* and *F. meridonale*. This is the first report of *F. fujikuroi*, *F. meridionale* and *F. asiaticum* causing CFSR in China.

**Table 3:** The disease index values for maize sheaths inoculated with *Fusarium* spp. in different maize cultivars.

*Fusarium* spp.	Isolate ID	Chuandan 455	Zhenghong 6	Chuandan 428	Ruiyu 16	Average
*F. proliferatum*	ynx8-3	34.44 ± 2.35ab	30.00 ± 2.62a	30.00 ± 1.44a	26.67 ± 1.91ab	30.28 ± 0.23a
	wx5-2	32.22 ± 2.91bc	32.22 ± 1.36a	28.89 ± 2.26ab	28.89 ± 1.09ab	
	dz1-1	37.78 ± 2.72a	28.89 ± 2.18ab	27.78 ± 2.17ab	25.56 ± 0.48b	
*F. fujikuroi*	× 4–3	30.00 ± 2.89cde	28.89 ± 2.25ab	25.56 ± 1.71b	28.89 ± 3.27ab	26.14 ± 2.58b
	cqw2-1	27.78 ± 0.96def	30.00 ± 3.53a	26.67 ± 2.27ab	30.00 ± 1.04a	
	w2-2–2	31.11 ± 3.00bcd	25.56 ± 3.25b	22.22 ± 3.45c	30.00 ± 2.20a	
*F. equiseti*	dj2-2	25.56 ± 1.61 fg	21.11 ± 0.75c	21.11 ± 1.20c	18.89 ± 1.09d	21.48 ± 1.14c
	w1-1–1	22.22 ± 0.56gh	18.89 ± 1.02 cd	22.22 ± 1.20c	16.67 ± 1.65de	
	cqws7-5	26.67 ± 0.65ef	25.56 ± 1.56b	16.67 ± 2.42d	22.22 ± 1.90c	
*F. verticillioides*	cqw4-2	16.67 ± 1.53ijk	18.89 ± 1.12 cd	13.33 ± 1.47defg	18.89 ± 2.61d	16.20 ± 0.57d
	× 4–5	18.89 ± 1.09hi	15.56 ± 1.65def	14.44 ± 1.43def	15.56 ± 0.72def	
	ms7-1	22.22 ± 0.78gh	13.33 ± 2.42efg	15.56 ± 1.40de	11.11 ± 0.37ghi	
*F. meridionale*	w2-2–1	15.56 ± 1.62ijk	15.56 ± 1.40def	8.89 ± 0.73hi	16.67 ± 1.70de	14.07 ± 0.35de
	cqw14-1	17.78 ± 0.73ij	14.44 ± 1.44efg	11.11 ± 1.20fghi	14.44 ± 1.44efg	
	w3-2	18.89 ± 1.10hi	13.33 ± 0.53efg	11.11 ± 0.43fghi	11.11 ± 0.67ghi	
*F. asiaticum*	cqw4-3	17.78 ± 1.54ij	16.67 ± 1.71de	12.22 ± 0.92efgh	13.33 ± 1.50efgh	13.80 ± 1.02de
	cqws4-4	16.67 ± 2.56ijk	15.56 ± 1.54def	12.22 ± 1.32efgh	11.11 ± 1.11ghi	
	yn1-2	15.56 ± 1.04ijk	12.22 ± 0.78fgh	10.00 ± 0.80ghi	12.22 ± 1.44fghi	
*F. graminearum*	w1-1–4	16.67 ± 1.15ijk	13.33 ± 0.93efg	7.78 ± 0.43i	11.11 ± 1.49ghi	11.57 ± 0.57ef
	clj2-1	14.44 ± 1.06jk	12.22 ± 1.09fgh	10.00 ± 0.65ghi	10.00 ± 1.43hi	
	wx6-1	14.44 ± 1.91jk	11.11 ± 0.20gh	8.89 ± 1.37hi	8.89 ± 1.18i	
*F. oxysporum*	× 1–4	13.33 ± 0.61kl	11.11 ± 1.19gh	7.78 ± 0.63i	12.22 ± 0.79fghi	10.14 ± 0.97f.
	c7-3	10.01 ± 1.08 l	8.89 ± 0.82 h	8.90 ± 0.88hi	8.90 ± 1.18i	

Different lowercase in the same column shows a significant difference at the level of p = 0.05, according to Duncan’s least significant range test.

## Discussion

Many studies have demonstrated that various *Fusarium* species, such as pathogens isolated in Henan, Hebei, Shandong and Gansu are associated with CFSR, which has significantly affected the quality and quantity of maize since it was first reported in northeast China in 2008^11,18^. In the present survey, the occurrence of CFSR was commonly observed, with two primary types of disease symptoms detected in the field (Fig. 1), including small black spots with a linear distribution and wet blotches with a tawny or brown color, similar to that described by Zhai^11^. Based on a survey conducted from 2015 to 2018, the disease incidence was very high, at 49.59–84.18%, with an average of 66.83%, and the severity of the disease index ranged from 7.32 to 37.62, with an average of 19.43 in the Sichuan fields.

Previous studies demonstrated that a complex of five *Fusarium* species, including *F. proliferatum*, *F. verticillioides*, *F. equiseti*, *F. graminearum* and *F. orysporum*, cause CFSR^11,19,20^. In the present study, we identified eight *Fusarium* species based on morphological characteristics and phylogenetic analysis (*EF1-α*), including *F. verticillioides*, *F. proliferatum, F. fujikuroi*, *F. asiaticum*, *F. equiseti, F. meridionale, F. graminearum* and *F. oxysporum*, with observed isolation frequencies of 30.00, 22.67, 15.33, 7.33, 6.00, 5.33, 3.33 and 1.33%, respectively. Many studies have shown that *Fusarium* species are consistently isolated and identified in mixed infections with other *Fusarium* species or fungi on many crops in the field^21,22^. In the present study, *F. verticillioides* and *F. proliferatum* were consistently observed in mixed infections in CFSR on maize, with a mixed infection rate of 14.67%.

*Fusarium* *proliferatum* is a ubiquitous, polyphagous, highly adaptable fungal pathogen of different plant species that attacks plants both in the field and during postharvest storage, causing blights, rots, and wilts on maize, garlic, soybean, tomato and *Aloe vera*^7,19,23–26^. Interestingly, several studies have shown that *F. proliferatum* is also the predominant pathogen of some commercial crops, such as *Polygonatum cyrtonema*, date palm and *Cymbidium*^27–29^. *F. proliferatum* was observed as the dominant fungus in infected garlic bulbs, with a high disease incidence of 35.40%, and it was confirmed as the causal agent of dry rot in garlic postharvest^30^. *F. proliferatum* was also highly pathogenic, and significant symptoms were also observed 2 weeks after being inoculated on onion^31^. On soybean, *F. proliferatum* easily infected seeds, with an observed disease severity index of 43.33–49.16%^32^. In our present study, *F. proliferatum* exhibited the highest virulence among the evaluated species, with a disease index of 30.28 in four different maize varieties, which is consistent with results of previous studies^8,16^. Additionally, *F. proliferatum* was also widely distributed, with an isolation frequency of 24.82%. *F. equiseti*, *F. verticillioides* and *F. graminearum* were successively reported as pathogens in CFSR^11,33^. Interestingly, *F. fujikuroi*, *F. meridionale* and *F. asiaticum* species were recorded as causing CFSR for the first time in China in the present study (Fig. 4).

Several studies have shown that *F. fujikuroi* causes rice bakanae disease and ear and stalk rot in maize^34,35^. In our present study, *F. fujikuroi* was the primary pathogen of CFSR, and *F. verticillioides* and *F. proliferatum* also exhibited strong pathogenicity, with an isolation frequency of 16.79% and a disease index of 28.06. *F. graminearum* is perhaps the best-known pathogen for causing head blight in wheat and ear and stalk rot in maize^36,37^. *F. graminearum* was detected in ≥ 80% of all *Fusarium* head blight (FHB) samples, sometimes even 100%^38^. In addition, the frequency of *F. graminearum* isolated from ears ranged from 30 to 71% with an average of 57%, and from stalks ranged from 43 to 81%, with the average of 65%^39^. Although the isolation frequency of *F. graminearum* from CFSR was only 3.65% in our present study, the potential risk of mycotoxins produced by *F. graminearum* to human health cannot be ignored^40^.

*Fusarium meridonale* is a member of the FGSC that is well-known to cause FHB in wheat and barley worldwide^41^. In addition, *F. meridionale* was also recently reported as pathogen in root rot on soybean under monoculture and ear rot on maize^42,43^. Moreover, *F. asiaticum* has been reported as a major and dominant causal agent of FHB on wheat and barley in China^44–46^ and was also detected as a pathogen causing *Gibberella* ear rot of maize and seedborne diseases of soybean^47,48^. Although *F. meridonale* and *F. asiaticum* were not predominant species in the present study, with low isolation frequencies, they were reported in China for the first time. On the other hand, *F. meridonale* and *F. asiaticum* also exhibited typical pathogenicity, with disease indices of 13.89 and 13.80, respectively.

In summary, in the present study, eight species of *Fusarium* were recovered from maize fields in Sichuan Province, China. Interestingly, three species, *F. fujikuroi*, *F. meridionale* and *F. asiaticum*, were reported to cause CFSR in China for the first time. All isolates could infect the maize sheath and had disease indices ranging from 10.14 to 30.28. *F. proliferatum* and *F. fujikuroi* were the primary pathogens of CFSR, exhibiting high isolation frequencies and disease indices. The results of the present study provides the theoretical basis for integrated control of CFSR in Sichuan Province of China.

## Methods

### Survey and sampling

Twenty-one county-level regions of 12 municipal administrations were surveyed for sampling between 2015 and 2018 in Sichuan, China. The disease percentage and severity of CFSR under natural conditions were investigated as described by Huang et al.^49^. Four fields that were at least 1 km apart were contained in each area, and five sites were evaluated in each field, with 100 ear leaf sheaths sampled per site. In addition, 150 diseased maize sheaths with dark brown spots or bronzing and tawny blotch were collected in valve bags and stored in a large ice box before cultural isolation.

### Isolated and morphological observations of *Fusarium* spp.

The pathogens were isolated from maize sheaths by tissue and single spore isolation^50,51^. Maize sheaths were cut into 0.5 cm × 0.5 cm pieces at the junction between disease and healthy tissue prior to isolation. The sheaths were surface-sterilized in 1% sodium hypochlorite for 1 min, rinsed extensively with sterilized water, soaked in 75% ethanol for 30 s, and then rinsed extensively with sterilized water again. Subsequently, all sample pieces were placed on potato dextrose agar (PDA; 200 g·L^−1^ potato, 10 g·L^−1^ glucose anhydrous, and 15 g·L^−1^ agar) plates supplemented with 0.2 g chloramphenicol for fungal isolation for 3 days at approximately 25 °C under 12-h lighting.

Additionally, all strains were transferred to liquid carboxymethyl cellulose medium (CMC; 7.5 g·L^−1^ carboxymethyl cellulose sodium, 0.5 g·L^−1^ yeast extract, 2.5 g·L^−1^ K_2_HPO_4_, and 0.25 g·L^−1^ MgSO_4_·7H_2_O)^52^. Then, the inoculated cultures were incubated for 5 days in a shaking incubator at 27 °C, 120 rpm. The culture characteristics and conidia of *Fusarium* species were morphologically assessed under a light microscope (Axio Imager Z2, ZEISS, Germany). One hundred thirty-seven isolates from 6 groups were identified as *Fusarium* spp.^53,54^.

### PCR amplification of *rDNA-ITS *and *EF-1α* sequences

All 137 obtained isolates were sub-cultured on PDA for 10 days at 25 °C with 12-h lighting. Approximately 20 mg of mycelia of each isolate was then scraped from the PDA plates with a sterilized ladle, and mycelia were ground to a powder in liquid nitrogen with a mortar. DNA was extracted using the cetyl-trimethylammonium bromide (CTAB) method^55^, and DNA concentration and quality were estimated using a Thermo Scientific NanoDrop 2000 Spectrophotometer (Massachusetts, USA) with the default setting for DNA assays.

PCR amplification of *rDNA-ITS*^56^ was performed with PCR primers ITS1 and ITS4 using the amplification conditions described by Schoch et al.^57^. Amplification of the *Fusarium* translation elongation factor 1α (*EF1-α*) gene was performed with the primer pair EF1 and EF2 using the amplification conditions described by O'Donnell et al.^58^. Molecular identification of *Fusarium* species was confirmed by PCR amplification using the primers ITS1 (TCCGTAGGTGAACCTGCGG) and ITS4 (GCTGCGTTCTTCATCGATGC) for the partial *rDNA-ITS* gene and the primers EF1-728F (CATCGAGAAGTTCGAGAAGG) and EF4-986R (TACTTGAAGGAACCCTTACC) for the partial *EF1-*α gene. PCR amplification was performed in a final volume of 25 μL containing 12.5 μL of 2 × PCR Master Mix (Vazyme, Nanjing, China), 0.5 μM of each primer and 10 ng of genomic DNA. The thermocycling conditions used for PCR amplification were as follows: 4 min at 94 °C followed by 35 cycles of 45 s at 94 °C, 60 s at 53 °C (for *EF1-α*) or 58 °C (for *rDNA-ITS*), and 1 min at 72 °C, with a final extension at 72 °C for 10 min. PCR products were detected by 1.5% agarose gel electrophoresis and then sequenced with an ABI-PRISM3730 automatic sequencer (Applied Biosystems, Foster, CA, USA) by Sangon Biotech Co., Ltd. (Shanghai, China).

### Phylogenetic analyses

Sequence analysis of the *rDNA-ITS* region was first performed using BLAST at the National Center for Biotechnology Information (NCBI) database (http://www.ncbi.nlm.nih.gov). Then, the *EF-1α* sequences from *Fusarium* species were compared to those in the NCBI database using the DNA BLAST program and the FUSARIUM-ID database (http://isolate.fusariumdb.org/guide.php). The sequences were aligned using the software ClustalX 2^59^. Phylogenetic analyses were performed using MEGA5 software package with the default parameters^60^. The alignments were manually edited to delete trimmed regions and discard incomplete sequences. Phylogenetic trees for each genomic region and their tandem sequences were constructed using the neighbor-joining (NJ) approach^61^ with 1000 bootstrap repeats and the pairwise deletion option^62^. *Bipolaris oryzae* was used as an outgroup (GenBank accession no. KJ 939510).

### Pathogenicity tests

Twenty-three isolates from eight identified species (3 representative isolates from each species, except for *F. oxysporum* with only 2 isolates) were assessed for pathogenicity on maize during the flowering period in 2016–2017. Subsequently, they were cultured on PDA medium at 25 °C under 12 h of ambient room lighting per day for 5 days. Thereafter, five mycelial plugs of each strain (5 mm in diameter) were transferred to 100 mL of CMC liquid medium and cultured for 3 days in a shaker incubator (25 °C, 160 rpm). The conidial suspensions were then filtered and adjusted to a final concentration of 1 × 10^5^ spores/mL. Four maize cultivars (Chuandan 455, Chuandan 428, Zhenghong 6 and Ruiyu 16) were sown in field plots, with each field plot containing 23 × 4 lines and 40 plants per line. Then, a 2-mL aliquot of a conidial suspension for each strain was injected into the first sheath above the ear leaf in 20 plants of each maize cultivar, and 20 control plants treated following the same procedure but were inoculated with sterile water^63^. Disease symptoms were assessed 25 days after inoculation, and disease severity was scored based on the average severity in 30 plants as described by Huang et al.^49^. Then, the disease index was calculated based on the average for each species. To confirm Koch’s postulates, isolation from maize sheaths infected with one representative isolate of each *Fusarium* species was attempted. Subsequently, symptomatic maize sheath tissue was sectioned, and 0.5-cm pieces were placed on PDA for reisolation under the same culture conditions.

### Data analysis

Differences in the field survey, growth rate, conidial length and width and pathogenicity were analyzed using Statistical Package for Social Sciences (SPSS) (version 22.0 for Windows). Analysis of variance was performed using the general linear model, and means were compared using Duncan’s New Multiple Range test with SPSS, with differences considered significant at P ≤ 0.05.

## Supplementary Information

Supplementary Table 1.

Supplementary Table 2.
