# Supplementary material for: Symptoms and pathogens diversity of Corn Fusarium sheath rot in Sichuan Province, China
Source: Sci Rep. 2021 Feb 2;11:2835. doi: 10.1038/s41598-021-82463-2 (PMC7854677; doi:10.1038/s41598-021-82463-2)
Supplement: Supplementary file 1 — Supplementary Table 1. [file 41598_2021_82463_MOESM1_ESM.docx]

**Symptoms and pathogens** [**diversity**](javascript:;) **of Corn *Fusarium* sheath rot in Sichuan Province, China**

**Wei Wang^1^, Bo Wang^1^, Xiaofang Sun^1^, Xiaobo Qi^1^, Conghao, Zhao^1^, Xiaoli Chang^1^, Muhammad Ibrahim Khaskheli^2^, Guoshu Gong^1^**^🖂^

**Table S1.** *Fusarium* species identified on the basis of *Fusarium* translation elongation factor 1α (*EF1-α*) sequences following their collection from maize sheaths from different areas in Sichuan, China.

| **Strain No.^1^** | **Species** | **Origin** | **Accession No.** |  | **Strain No.^1^** | **Species** | **Origin** | **Accession No.** |
| --- | --- | --- | --- | --- | --- | --- | --- | --- |
| yn1-2 | *Fusarium asiaticum* | Yucheng, Ya’an, China | MH448748 |  | prp7-1 | *Fusarium proliferatum* | Renhe, Panzhihua, China | MH448831 |
| yn1-1-1 | *Fusarium asiaticum* | Yucheng, Ya’an, China | MH448749 |  | ynx10-2 | *Fusarium proliferatum* | Yucheng, Ya’an, China | MH448809 |
| cqw4-3 | *Fusarium asiaticum* | Chongzhou, Chengdu, China | MH448750 |  | prp2-1 | *Fusarium proliferatum* | Renhe, Panzhihua, China | MH448807 |
| cqw22-1-1 | *Fusarium asiaticum* | Chongzhou, Chengdu, China | MH448751 |  | prp10-3 | *Fusarium proliferatum* | Renhe, Panzhihua, China | MH448832 |
| cqwj1-1-2 | *Fusarium asiaticum* | Chongzhou, Chengdu, China | MH448752 |  | prpx2-2 | *Fusarium proliferatum* | Renhe, Panzhihua, China | MH448833 |
| cqws4-4 | *Fusarium asiaticum* | Chongzhou, Chengdu, China | MH448753 |  | ynx4-1 | *Fusarium proliferatum* | Yucheng, Ya’an, China | MH448834 |
| x3-2 | *Fusarium asiaticum* | Xindu, Chengdu, China | MH448754 |  | wx5-1 | *Fusarium proliferatum* | Wenjiang, Chengdu, China | MH448835 |
| x3-4 | *Fusarium asiaticum* | Xindu, Chengdu, China | MH448755 |  | wx8-4 | *Fusarium proliferatum* | Wenjiang, Chengdu, China | MH448808 |
| x3-10 | *Fusarium asiaticum* | Xindu, Chengdu, China | MH448756 |  | prp2-2-1 | *Fusarium proliferatum* | Renhe, Panzhihua, China | MH448806 |
| ynx8-1 | *Fusarium asiaticum* | Yucheng, Ya’an, China | MH448757 |  | wx6-2 | *Fusarium proliferatum* | Wenjiang, Chengdu, China | MH448836 |
| dzx12-1 | *Fusarium asiaticum* | Zhongjiang, Deyang, China | MH448758 |  | wx6-5 | *Fusarium proliferatum* | Wenjiang, Chengdu, China | MH448837 |
| cqws7-5 | *Fusarium equiseti* | Chongzhou, Chengdu, China | MH448791 |  | wx8-3 | *Fusarium proliferatum* | Wenjiang, Chengdu, China | MH448838 |
| w1-1-1 | *Fusarium equiseti* | Wenjiang, Chengdu, China | MH448790 |  | ynx8-5 | *Fusarium proliferatum* | Yucheng, Ya’an, China | MH448839 |
| cqwx2-6 | *Fusarium equiseti* | Chongzhou, Chengdu, China | MH448787 |  | dzx8-1 | *Fusarium proliferatum* | Zhongjiang, Deyang, China | MH448840 |
| dj1-2 | *Fusarium equiseti* | Dayi, Chengdu, China | MH448792 |  | ms4 | *Fusarium verticillioides* | Dongpo, Meishan, China | MH448843 |
| dj2-2 | *Fusarium equiseti* | Dayi, Chengdu, China | MH448793 |  | cqw13-2 | *Fusarium verticillioides* | Chongzhou, Chengdu, China | MH448844 |
| dj2-3 | *Fusarium equiseti* | Dayi, Chengdu, China | MH448794 |  | cqwx4-1 | *Fusarium verticillioides* | Chongzhou, Chengdu, China | MH448845 |
| dj2-1 | *Fusarium equiseti* | Dayi, Chengdu, China | MH448795 |  | ms8-2 | *Fusarium verticillioides* | Dongpo, Meishan, China | MH448846 |
| q4-3 | *Fusarium equiseti* | Qionglai, Chengdu, China | MH448789 |  | ms2-4 | *Fusarium verticillioides* | Dongpo, Meishan, China | MH448847 |
| q1-4 | *Fusarium equiseti* | Qionglai, Chengdu, China | MH448788 |  | jy2-1 | *Fusarium verticillioides* | Jianyang, Chengdu, China | MH448848 |
| x4-3 | *Fusarium fujikuroi* | Xindu, Chengdu, China | MH448765 |  | cqw21-1 | *Fusarium verticillioides* | Chongzhou, Chengdu, China | MH448849 |
| x4-7 | *Fusarium fujikuroi* | Xindu, Chengdu, China | MH448766 |  | cqw4-2 | *Fusarium verticillioides* | Chongzhou, Chengdu, China | MH448850 |
| yb1-2 | *Fusarium fujikuroi* | Yucheng, Ya’an, China | MH448767 |  | cqw24-1 | *Fusarium verticillioides* | Chongzhou, Chengdu, China | MH448851 |
| dz3-1 | *Fusarium fujikuroi* | Zhongjiang, Deyang, China | MH448768 |  | x4-5 | *Fusarium verticillioides* | Xindu, Chengdu, China | MH448852 |
| cqw2-1 | *Fusarium fujikuroi* | Chongzhou, Chengdu, China | MH448769 |  | x4-12 | *Fusarium verticillioides* | Xindu, Chengdu, China | MH448853 |
| cqw23-1 | *Fusarium fujikuroi* | Chongzhou, Chengdu, China | MH448770 |  | cqw1-2 | *Fusarium verticillioides* | Chongzhou, Chengdu, China | MH448854 |
| cqw1-4 | *Fusarium fujikuroi* | Chongzhou, Chengdu, China | MH448771 |  | c8-1 | *Fusarium verticillioides* | Chongzhou, Chengdu, China | MH448855 |
| cqws2-3-2 | *Fusarium fujikuroi* | Chongzhou, Chengdu, China | MH448772 |  | ms8-1 | *Fusarium verticillioides* | Dongpo, Meishan, China | MH448856 |
| clj2-5 | *Fusarium fujikuroi* | Chongzhou, Chengdu, China | MH448773 |  | cqws2-3 | *Fusarium verticillioides* | Chongzhou, Chengdu, China | MH448857 |
| dw4 | *Fusarium fujikuroi* | Dayi, Chengdu, China | MH448774 |  | cq3 | *Fusarium verticillioides* | Chongzhou, Chengdu, China | MH448858 |
| cqws10-4 | *Fusarium fujikuroi* | Chongzhou, Chengdu, China | MH448775 |  | cqwx4-3 | *Fusarium verticillioides* | Chongzhou, Chengdu, China | MH448859 |
| cqws1-3 | *Fusarium fujikuroi* | Chongzhou, Chengdu, China | MH448776 |  | w1-1-2 | *Fusarium verticillioides* | Wenjiang, Chengdu, China | MH448860 |
| cqws10-1 | *Fusarium fujikuroi* | Chongzhou, Chengdu, China | MH448777 |  | y3-5 | *Fusarium verticillioides* | Yucheng, Ya’an, China | MH448861 |
| cqws10-2 | *Fusarium fujikuroi* | Chongzhou, Chengdu, China | MH448778 |  | y3-6 | *Fusarium verticillioides* | Yucheng, Ya’an, China | MH448862 |
| clj2-3 | *Fusarium fujikuroi* | Chongzhou, Chengdu, China | MH448780 |  | dz9-2 | *Fusarium verticillioides* | Zhongjiang, Deyang, China | MH448863 |
| clj2-2 | *Fusarium fujikuroi* | Chongzhou, Chengdu, China | MH448786 |  | ms7-1 | *Fusarium verticillioides* | Dongpo, Meishan, China | MH448864 |
| y3-3 | *Fusarium fujikuroi* | Yucheng, Ya’an, China | MH448785 |  | x4-4 | *Fusarium verticillioides* | Xindu, Chengdu, China | MH448865 |
| clj1-1 | *Fusarium fujikuroi* | Chongzhou, Chengdu, China | MH448779 |  | ms7-4 | *Fusarium verticillioides* | Dongpo, Meishan, China | MH448866 |
| w2-2-2 | *Fusarium fujikuroi* | Wenjiang, Chengdu, China | MH448764 |  | x3-7 | *Fusarium verticillioides* | Xindu, Chengdu, China | MH448867 |
| wx7-1 | *Fusarium fujikuroi* | Wenjiang, Chengdu, China | MH448782 |  | dzx2-1 | *Fusarium verticillioides* | Zhongjiang, Deyang, China | MH448868 |
| ynx8-2 | *Fusarium fujikuroi* | Yucheng, Ya’an, China | MH448781 |  | prp2-4 | *Fusarium verticillioides* | Renhe, Panzhihua, China | MH448841 |
| dzx9-2 | *Fusarium fujikuroi* | Zhongjiang, Deyang, China | MH448784 |  | prpx3 | *Fusarium verticillioides* | Renhe, Panzhihua, China | MH448869 |
| ynx1-1 | *Fusarium fujikuroi* | Yucheng, Ya’an, China | MH448783 |  | prpx3-1 | *Fusarium verticillioides* | Renhe, Panzhihua, China | MH448842 |
| clj2-1 | *Fusarium graminearum* | Chongzhou, Chengdu, China | MH448759 |  | cqn3-1 | *Fusarium verticillioides* | Chongzhou, Chengdu, China | MH448870 |
| w1-1-4 | *Fusarium graminearum* | Wenjiang, Chengdu, China | MH448760 |  | dzx4-3 | *Fusarium verticillioides* | Zhongjiang, Deyang, China | MH448871 |
| w1-1-3 | *Fusarium graminearum* | Wenjiang, Chengdu, China | MH448761 |  | prp3-2 | *Fusarium verticillioides* | Renhe, Panzhihua, China | MH448872 |
| ynx3-1 | *Fusarium graminearum* | Yucheng, Ya’an, China | MH448762 |  | prp7-2 | *Fusarium verticillioides* | Renhe, Panzhihua, China | MH448873 |
| wx6-1 | *Fusarium graminearum* | Wenjiang, Chengdu, China | MH448763 |  | wx8-1 | *Fusarium verticillioides* | Wenjiang, Chengdu, China | MH448874 |
| cqwx2-2 | *Fusarium meridionale* | Chongzhou, Chengdu, China | MH448796 |  | cqn1-2 | *Fusarium verticillioides* | Chongzhou, Chengdu, China | MH448875 |
| w1-2-1 | *Fusarium meridionale* | Wenjiang, Chengdu, China | MH448797 |  | cqn2 | *Fusarium verticillioides* | Chongzhou, Chengdu, China | MH448876 |
| w2-2-1 | *Fusarium meridionale* | Wenjiang, Chengdu, China | MH448798 |  | wx1-1 | *Fusarium verticillioides* | Wenjiang, Chengdu, China | MH448877 |
| ybc2-1 | *Fusarium meridionale* | Cuiping, Yibin, China | MH448799 |  | dzx1-6 | *Fusarium verticillioides* | Zhongjiang, Deyang, China | MH448878 |
| cqw14-1 | *Fusarium meridionale* | Chongzhou, Chengdu, China | MH448800 |  | dzx3-1 | *Fusarium verticillioides* | Zhongjiang, Deyang, China | MH448879 |
| cqwj1-2-3 | *Fusarium meridionale* | Chongzhou, Chengdu, China | MH448801 |  | dzx12-4 | *Fusarium verticillioides* | Zhongjiang, Deyang, China | MH448880 |
| w3-2 | *Fusarium meridionale* | Wenjiang, Chengdu, China | MH448802 |  | dzx11-1 | *Fusarium verticillioides* | Zhongjiang, Deyang, China | MH448881 |
| wx3-1 | *Fusarium meridionale* | Wenjiang, Chengdu, China | MH448803 |  | dzx11-4 | *Fusarium verticillioides* | Zhongjiang, Deyang, China | MH448882 |
| x1-4 | *Fusarium oxysporum* | Xindu, Chengdu, China | MH448805 |  | wx6-4 | *Fusarium verticillioides* | Wenjiang, Chengdu, China | MH448883 |
| c7-3 | *Fusarium oxysporum* | Chongzhou, Chengdu, China | MH448804 |  | cqn1-1 | *Fusarium verticillioides* | Chongzhou, Chengdu, China | MH448884 |
| w2-1-1 | *Fusarium proliferatum* | Wenjiang, Chengdu, China | MH448811 |  | dzx8-2 | *Fusarium verticillioides* | Zhongjiang, Deyang, China | MH448885 |
| y3-2 | *Fusarium proliferatum* | Yucheng, Ya’an, China | MH448812 |  | NRRL22944 | *Fusarium proliferatum* | Germany | AF160280^1^ |
| dz4-2-2 | *Fusarium proliferatum* | Zhongjiang, Deyang, China | MH448813 |  | NRRL13566 | *Fusarium fujikuroi* | Taiwan, China | AF160279^1^ |
| dz1-1 | *Fusarium proliferatum* | Zhongjiang, Deyang, China | MH448814 |  | NRRL66432 | *Fusarium fujikuroi* | USA | KX656194^2^ |
| ybc2-3 | *Fusarium proliferatum* | Cuiping, Yibin, China | MH448815 |  | NRRL66438 | *Fusarium fujikuroi* | USA | KX656196^2^ |
| w2-2-3 | *Fusarium proliferatum* | Wenjiang, Chengdu, China | MH448816 |  | NRRL22902 | *Fusarium oxysporum* | USA | AF160312^1^ |
| n22-1 | *Fusarium proliferatum* | Weiyuan, Neijiang, China | MH448817 |  | NRRL15111 | *Fusarium verticillioides* | USA | JF740737^3^ |
| ms4-3-1 | *Fusarium proliferatum* | Dongpo, Meishan, China | MH448818 |  | NRRL22172 | *Fusarium verticillioides* | Germany | AF160262^1^ |
| x5-2 | *Fusarium proliferatum* | Xindu, Chengdu, China | MH448820 |  | NRRL26419 | *Fusarium iequiseti* | Germany | GQ505599^4^ |
| ynx6-1 | *Fusarium proliferatum* | Yucheng, Ya’an, China | MH448821 |  | NRRL36466 | *Fusarium equiseti* | Denmark | GQ505653^4^ |
| ynx8-3 | *Fusarium proliferatum* | Yucheng, Ya’an, China | MH448822 |  | NRRL52760 | *Fusarium meridionale* | Costa Rica | JF740835^3^ |
| ynx8-4 | *Fusarium proliferatum* | Yucheng, Ya’an, China | MH448810 |  | NRRL34352 | *Fusarium meridionale* | USA | EF428656^5^ |
| ynx9-1 | *Fusarium proliferatum* | Yucheng, Ya’an, China | MH448823 |  | NRRL31622 | *Fusarium meridionale* | USA | EF428624^5^ |
| prp10-4 | *Fusarium proliferatum* | Renhe, Panzhihua, China | MH448824 |  | NRRL52929 | *Fusarium graminearum* | Turkey | JF740871^3^ |
| ynx4-2 | *Fusarium proliferatum* | Yucheng, Ya’an, China | MH448825 |  | NRRL52799 | *Fusarium graminearum* | Turkey | JF740867^3^ |
| wx5-2 | *Fusarium proliferatum* | Wenjiang, Chengdu, China | MH448826 |  | NRRL6101 | *Fusarium asiaticum* | Japan | AF212450^6^ |
| wx7-2 | *Fusarium proliferatum* | Wenjiang, Chengdu, China | MH448827 |  | NRRL26156 | *Fusarium asiaticum* | China | AF212452^6^ |
| prpx2-3 | *Fusarium proliferatum* | Renhe, Panzhihua, China | MH448828 |  | NRRL13818 | *Fusarium asiaticum* | Japan | AF212451^6^ |
| ynx9-1-1 | *Fusarium proliferatum* | Yucheng, Ya’an, China | MH448829 |  | B33 | *Bipolaris oryzae* | India | KJ939510 |
| wx5-3 | *Fusarium proliferatum* | Wenjiang, Chengdu, China | MH448830 |  |  |  |  |  |

^1^ The abbreviations for the culture collections: NRRL (National Center for Agricultural Utilization Research) Peoria, Illinois, USA.

Reference

1. Herron, D. A. *et al*. Novel taxa in the *Fusarium fujikuroi* species complex from *Pinus* spp. *Stud. Mycol.* **80**, 131-150 (2015).
2. Bolton, S. L., Brannen, P. M. & Glenn, A. E. A novel population of *Fusarium* *fujikuroi* isolated from Southeastern U.S. winegrapes reveals the need to re-evaluate the species’ fumonisin production. *Toxins* **8**, 254 (2016).
3. O’Donnell, K., Humber, R. A., Geiser, D. M., Kang, S. & Rehner, S. A. Phylogenetic diversity of insecticolous fusaria inferred from multilocus DNA sequence data and their molecular identification via FUSARIUM-ID and *Fusarium* MLST. *Mycologia* **104**, 427-445 (2012).
4. O’Donnell, K. *et al*. Novel multilocus sequence typing scheme reveals high genetic diversity of human pathogenic members of the *Fusarium* *incarnatum-F. equiseti* and *F. chlamydosporum* species complexes within the united states. *J. Clin. Microbiol.* **47**, 3851-3861 (2009).
5. Ward, T. J. *et al*. An adaptive evolutionary shift in *Fusarium* head blight pathogen populations is driving the rapid spread of more toxigenic *Fusarium graminearum* in North America. *Fungal Genet. Biol.* **45**, 473-484 (2008).
6. O’Donnell, K., Kistler, H. C., Tacke, B. K. & Casper, H. H. Gene genealogies reveal global phylogeographic structure and reproductive isolation among lineages of *Fusarium graminearum*, the fungus causing wheat scab. *PNAS* **97**, 7905-7910 (2000).
